# The "Coromandel"

**Published:** 1895-12-14

**Authors:** 


### "THE COROMANDEL."

### THE HOSPITAL SHIP FOR THE ASHANTI EXPEDITION.

The preparations made on board the P. and O. Company's steamer *Coromandel*—" Hospital and Transport Ship No. 7 " —chartered by the Admiralty for the reception of the sick and wounded in the event of a war with Ashanti, have been of the most complete kind. No boats can make more excellent transport ships than these huge ocean-going steamers, and those visitors privileged to inspect the arrangements on board the *Coromandel* cannot fail to have been struck by the comfort and extent of the accommodation.

The *Coromandel*, painted white from stem to stern, with a red line running along the sides, having on board the last body of troops to go out to the Gold Coast, with medical and nursing staff, left the Thames in the early hours of Sunday morning last. After conveying the Special Service Corps to Cape Coast Castle she will anchor some two miles from shore, and be used as hospital and sanatorium for troops invalided from the front. A great deal of structural alteration has of course been necessary to fit the boat for her present purpose. The second-class and a portion of the first-class saloons have been fitted up as a hospital, containing 140 cots—the latter so constructed as to swing with the rolling of the ship, while they may be secured firmly if desired. The wards are painted a soft, light shade of green, pleasant to the eye. Punkahs are arranged between the beds, and canvas screens, so that the wards may be kept as cool as possible in tropical weather. A new system of ventilation, whereby the exhausted air is extracted through shafts by steam, has been arranged; the electric light fitted everywhere, as well as arrangements for heating kettles, &c., so as to provide every convenience for the sick with a minimum of trouble. There are rooms for the manufacture of ice, a laundry, fitted with every requirement, and laundry men to attend to it; a soda water plant, and an extensive arrangement for the filtration of drinking-water, also under the care of a special attendant.

The ward arrangements are excellent, with a good supply of well-fitted bath-rooms and lavatories; surgery, dispensary, and operating-room are provided, all most conveniently disposed as regards position. The cooking for the hospital will be done apart. The catering is undertaken by the P. and O. Company, and it goes without saying that it is therefore certain to be thoroughly well done. The cabins for the medical and surgical staff are conveniently near the wards, and are most comfortable—even luxurious. The chief medical officer attached to the *Coromandel* is Brigade-Surgeon Lieutenant-Colonel Townsend, the others being Surgeon-Major R. Porter and Surgeon-Major J. R. Dodd, all of the Army Medical Staff. Two chaplains—Church of England and Roman Catholic—accompany the troops.

The nursing staff consists of three Sisters of the Army Nursing Service, Miss J. A. Gray (lady superintendent of the hospital of the Coldstream Guards) in charge, Sister M'Curdy, and Sister Potts. Sister Gray has had plenty of experience in war time. She is the senior nurse in the service, having commenced her hospital career in Liverpool more than 22 years ago. In 1873 she entered Netley Hospital, and first saw active service in the Zulu war in 1879. Again she was sent to Egypt in 1882, and nursed through the Egyptian war, and afterwards accompanied the Gordon Relief Expedition up the Nile to Wady Halfa, nursing the men through the cholera epidemic, and being absent from England for nearly four years. Since then Sister Gray has served at Woolwich and Portsmouth, and was in charge of the Coldstream Guards' hospital, as mentioned above, until summoned to go out on the present expedition. As decorations Sister Gray has had conferred on her the Zulu and Egyptian war medals, the Egyptian Cross, and, worn conspicuously by itself on the shoulder, the Royal Red Cross, bestowed for her active labours in Egypt.

The accommodation for Sister Gray and her nurses on board the *Coromandel* is most comfortable. Partitioned off by a heavy curtain, their quarters are quite to themselves, and consist of two sleeping cabins, one double-berthed, another cabin fitted as sitting-room for them when not on duty, bath-room and lavatory, and pantry, with ample means at hand in the way of electric kettles, for that tea-making dear to the heart of every nurse. Altogether, there would seem to be nothing left to seek on board the Ashanti hospital ship in the way of due forethought for the comfort alike of possible sick and wounded and of those under whose medical and nursing care they will be.

